# Subthalamic deep brain stimulation reduces pathological information transmission to the thalamus in a rat model of parkinsonism

**DOI:** 10.3389/fncir.2015.00031

**Published:** 2015-07-06

**Authors:** Collin J. Anderson, Daylan T. Sheppard, Rachel Huynh, Daria Nesterovich Anderson, Christian A. Polar, Alan D. Dorval

**Affiliations:** Department of Bioengineering, University of UtahSalt Lake City, UT, USA

**Keywords:** parkinson disease, neural information, deep brain stimulation, substantia nigra pars reticulata, 6-hydroxydopamine, thalamic nuclei

## Abstract

The degeneration of dopaminergic neurons in the substantia nigra pars compacta leads to parkinsonian motor symptoms via changes in electrophysiological activity throughout the basal ganglia. High-frequency deep brain stimulation (DBS) partially treats these symptoms, but the mechanisms are unclear. We hypothesize that motor symptoms of Parkinson’s disease (PD) are associated with increased information transmission from basal ganglia output neurons to motor thalamus input neurons and that therapeutic DBS of the subthalamic nucleus (STN) treats these symptoms by reducing this extraneous information transmission. We tested these hypotheses in a unilateral, 6-hydroxydopamine-lesioned rodent model of hemiparkinsonism. Information transfer between basal ganglia output neurons and motor thalamus input neurons increased in both the orthodromic and antidromic directions with hemiparkinsonian (hPD) onset, and these changes were reversed by behaviorally therapeutic STN-DBS. Omnidirectional information increases in the parkinsonian state underscore the detrimental nature of that pathological information and suggest a loss of information channel independence. Therapeutic STN-DBS reduced that pathological information, suggesting an effective increase in the number of independent information channels. We interpret these data with a model in which pathological information and fewer information channels diminishes the scope of possible motor activities, driving parkinsonian symptoms. In this model, STN-DBS restores information-channel independence by eliminating or masking the parkinsonism-associated information, and thus enlarges the scope of possible motor activities, alleviating parkinsonian symptoms.

## Introduction

Deep brain stimulation (DBS) is an accepted therapy for several neurological conditions (Benabid et al., 1998; Hariz et al., 2013). In particular, DBS of the subthalamic nucleus (STN) or globus pallidus internus (GPi) can alleviate motor symptoms of Parkinson’s disease (PD), including bradykinesia, rigidity, and tremor (Limousin-Dowsey et al., 1999; Lyons and Pahwa, 2004). These motor symptoms are associated with pathological neural activity in basal ganglia that propagates into the thalamocortical motor loop (Chesselet and Delfs, 1996; Galvan et al., 2015). Effective DBS modulates the neural activity in basal ganglia (Boraud et al., 1996; Benazzouz et al., 2000; Da Cunha et al., 2015) and consequently motor thalamus (Anderson et al., 2003; Hashimoto et al., 2003; Hershey et al., 2003), but the therapeutic mechanisms of this modulation are poorly understood.

Over the past two decades, the accepted model of pathological neural activity in PD has shifted from one based merely on excitation- and inhibition-driven changes in firing rate to one based on changes in firing patterns. Throughout the basal ganglia-thalamic circuit, neurons burst (Bergman et al., 1994; Magnin et al., 2000; Tang et al., 2005) and oscillate (Raz et al., 2000; Brown et al., 2004) more in the parkinsonian brain. Supporting this change in firing pattern model, therapeutic DBS reduces those bursts (Anderson et al., 2003; Grill et al., 2004; Tai et al., 2012) and oscillations (Meissner et al., 2005; McConnell et al., 2012). With others, we have argued that this PD-associated pathological activity constitutes substantial neurological disorder, and that DBS alleviates PD-symptoms by regularizing neural activity in computational (Rubin and Terman, 2004; Guo et al., 2008; Dorval et al., 2009; So et al., 2012), rodent (Degos et al., 2005; Dorval and Grill, 2014), non-human primate (Hashimoto et al., 2003; Bar-Gad et al., 2004; Dorval et al., 2008), and human studies (Dorval et al., 2010).

Neuronal disorder is readily quantified as firing pattern entropy, which bounds and contributes to information directed between neurons. This directed information measures the influence that one cell has on another cell. If disordered firing patterns contribute to parkinsonian symptoms, we hypothesize that they do so by transmitting pathological information from basal ganglia to downstream structures in the thalamo-cortical motor loop (Grill et al., 2004). To test this hypothesis experimentally, we used a rodent model of PD to examine neuronal information directed from basal ganglia efferents in substantia nigra pars reticulata (SNr), to the ventral anterior (VA) thalamus under healthy and hemiparkinsonian (hPD) conditions, and in the presence of behaviorally therapeutic STN-DBS. In hPD relative to control, we found large increases in information directed orthodromically (i.e., in the anterograde direction) from SNr to VA, and antidromically (i.e., in the retrograde direction) from VA to SNr. These hPD-associated informational increases were reversed entirely by therapeutic STN-DBS.

## Methods

All surgical and experimental procedures were performed at the University of Utah, approved by the Institutional Animal Care and Use Committee of the University of Utah, and complied with U.S. Public Health Service policy on care and use of laboratory animals.

### Experimental Procedures

The timeline of the protocol for five Long-Evans rats of both sexes weighing 225–325 g comprised the following: surgical implantation followed by a 2 week recovery period; 2–5 control recording sessions at least 1 day apart; 6-OHDA injection followed by a 2 week recovery period; and 4–8 experimental recording sessions at least 1 day apart; and for three rats, terminal perfusion, fixing, and brain removal.

#### Surgical Procedure

Rats were administered orally 10 mg of rimadyl (Bio-Serv, Flemington, NJ, USA) and, after consumption, anesthetized with two percent isoflurane. The surgical site was shaved and disinfected with isopropyl alcohol and povidone-iodine. Rats were placed on a heating pad in a stereotactic frame (Kopf Instruments, Tujunga, CA, USA), and an incision was made and opened to the skull. Craniotomy sites were measured and marked with respect to bregma (Paxinos and Watson, 2008). Seven titanium bone screws were inserted through burr holes in the skull to anchor the eventual acrylic cap to the skull. Stainless steel ground wires were attached to two of the anchor screws.

Neural implants included a cannula, 4-channel stimulating array, and two 16-channel recording arrays. To enable the eventual insertion of a needle to the medial forebrain bundle (MFB), a 23-gauge stainless steel cannula was implanted through a burr hole 1.0 mm anterior and 2.0 mm lateral, in the sagittal plane and angled 26.5° posteriorly from the coronal plane. A 4-channel micro-stimulating array—2 × 2 grid of 75 μm platinum-iridium electrodes (~100 kΩ @ 1 kHz) with 400 μm spacing (Microprobes for Life Science, Inc., Gaithersburg, MD, USA)—was implanted vertically into the STN (3.6 mm posterior, 2.6 mm lateral, and 7.8 mm ventral). Medial electrodes were 500 μm longer than their lateral counterparts to match STN anatomy. Sixteen-channel micro-recording arrays—4 × 4 grid of 20 μm stainless steel electrodes (~1.0 MΩ @ 1 kHz) with 400 μm spacing (ibid) were implanted into VA and SNr. The VA array reached its target (2.1 mm posterior, 1.9 mm lateral, and 6.2 mm ventral) by advancing 6.3 mm through a burr hole 1.1 mm posterior and 1.9 mm lateral, in the sagittal plane and angled 9.0° posteriorly from the coronal plane. The SNr array reached its target (5.5 mm posterior, 2.3 mm lateral, and 7.7 mm ventral) by advancing 7.8 mm through a burr hole 6.5 mm posterior and 2.3 mm lateral, in the sagittal plane and angled 7.4° anteriorly from the coronal plane.

After each hardware element was implanted, a layer of dental acrylic was applied to attach it firmly to at least one of the anchor screws. Following all implantations, ground contacts of the recording arrays were tied to wires on the ground screws. The screws, cannula, and arrays were encased in a thick dental acrylic cap, built smoothly to include no sharp edges or points. The incision was sutured around the acrylic head-cap, with triple antibiotic ointment applied inside and outside of the wound to prevent infection. Rats were administered the analgesic ketoprofen (5 mg/kg) via intraperitoneal injection, monitored for 2 h, and allowed to recover for 14 days prior to recordings.

#### Unilateral Dopamine Lesion

After completion of both behavioral and electrophysiological recordings (see below), rats were anesthetized with two percent isoflurane and given intraperitoneal injections of desipramine hydrochloride (25 mg/kg) and pargyline hydrochloride (50 mg/kg) before being placed in the same stereotactic frame. A 32-gauge needle (Hamilton Co., Reno, NV, USA) was inserted through the angled cannula 6.7 mm beyond the cranial surface, to the MFB target (2.0 mm posterior, 2.0 mm lateral, 6.0 mm ventral). The needle was inserted an additional 0.5 mm, left for 5 min to create a pocket, and then retracted by 0.5 mm. Thirty minutes after the intraperitoneal injections, 8.0 μg of 6-OHDA dissolved in 8.0 μl of saline were injected at a rate of 1.0 μl/min. Ten minutes after injection, the needle was withdrawn at a speed not exceeding 1.0 mm/min. Rats were returned to their home cages for 14 days before recording under hPD and STN-DBS conditions.

#### Deep Brain Stimulation

To provide DBS to the STN, rats were briefly anesthetized with two percent isoflurane and a 4-channel tether was attached to the connector of the micro-stimulating array on the head. Rats were quickly moved to the recording chamber before awaking, and the other end of the tether was connected to an analog stimulator (Grass Technologies, Warwick, RI, USA). Bipolar stimulation was delivered through the micro-stimulating array, with both medial contacts set as anode and both lateral contacts set as cathode. The stimulation waveform consisted of symmetric, 100 μs biphasic current pulses repeating at 100 Hz. Two weeks following 6-OHDA injection, stimulation thresholds were determined by gradually increasing stimulation amplitude until threshold, denoted by the appearance of physical side effects (~200 μA). The DBS-associated side effects included atypical whisker twitching, other dystonic facial contractions, or repetitive rotations contralateral to stimulation. This process was repeated three times for each animal to determine a truly minimal threshold. Therapeutic DBS amplitude was then set to 90% of the minimum side-effect threshold. While stimulation was applied only during DBS trials, the tether was connected during some healthy-control and hPD trials to ensure that the tether did not induce significant changes in either behavior or electrophysiological activity.

#### Behavioral Recording Protocol

In four of five rats, left-side vs. right-side body asymmetry was quantified using a custom rat tracking system. Rats were placed in a darkened 30 × 45 cm box and recorded from above with an infrared camera for 60 min. In real time, custom software differentiated the dark coat of the rat from the white bedding, and calculated rat position and orientation within the box. Rats were typically untethered during healthy and hPD trials, but were necessarily tethered during DBS trials. Subsequent to the behavioral sessions, data files containing rat location and orientation were analyzed to quantify the leftward and rightward turns the rat made in each trial.

A fifth rat completed the study before completion of the rat tracking system. For this rat, a standard dextroamphetamine-induced rotation protocol was used to verify an increase or decrease of left-side vs. right-side body asymmetry under each condition (Maesawa et al., 2004). Briefly, under each condition, 5 mg/kg of dextroamphetamine was injected intraperitoneally. The rat was placed in its home cage, and complete rotations in both directions were counted manually for 30 min. An hPD condition was identified when the rat performed at least 10 more ipsilateral rotations than contralateral rotations per minute; an hPD-alleviated condition, produced by STN-DBS, was identified when the rat performed at most six more ipsilateral rotations than contralateral rotations per minute.

#### Electrophysiological Recording Protocol

Rats were briefly anesthetized with two percent isoflurane and the connectors of their micro-recording arrays were attached to a pair of 16-channel head stages and associated tethers to a multichannel stimulation and recording system (SnR, Alpha Omega, Alpharetta, GA, USA). For STN-DBS trials, rats were additionally connected to an analog stimulator with a 4-channel tether, as described above. Ten minutes after waking in their home cages with lids removed, electrophysiological recordings began and lasted 15 min. Local field potentials (LFPs) and threshold-crossing segmented data were referenced to transcranial ground screws and recorded simultaneously from both SNr and VA. Segmented data thresholds were chosen to include action potential traces, while minimizing noise and low amplitude multiunit activity.

#### Histological Analysis

Of five rats in the study, the head-caps of two dislodged before the target number of hPD and DBS trials (five each) had been completed, and those rats were euthanized without perfusion. The remaining three rats were perfused with phosphate buffer solution and fixed with four percent paraformaldehyde. Brains were immediately removed and soaked in the same formalin. After 24 h, brains were transferred to phosphate buffer solution, sectioned into 200 μm slices, and set onto slides. After 24 h of drying, slides including STN, SNr or VA were stained with cresyl violet by standard methods. Gross anatomical analysis verified all electrode locations as being within SNr, VA, and STN.

### Data Analyses

Analyses were performed on standard computers. Unit spike sorting was done in Plexon Offline Sorter (Plexon, Inc., Dallas, TX, USA) and all other analyses were done in MATLAB (The MathWorks, Natick, MA, USA).

#### Behavioral Analysis

Rat position and orientation during behavioral trials were recorded by custom tracking software at 5–10 samples/s. We integrated the total degrees rotated to the right (θ_R_) and to the left (θ_L_) during each 60 min trial. Given an injection of 6-OHDA to the right MFB, symptoms of hPD should present on the left side of the body, and the rats should rotate to the right, ipsilateral to injection. Ipsilateral rotation preference was calculated as: 100% × θ_R_ / (θ_L_+ θ_R_).

#### Spike Train Analysis

**Figure 1:**
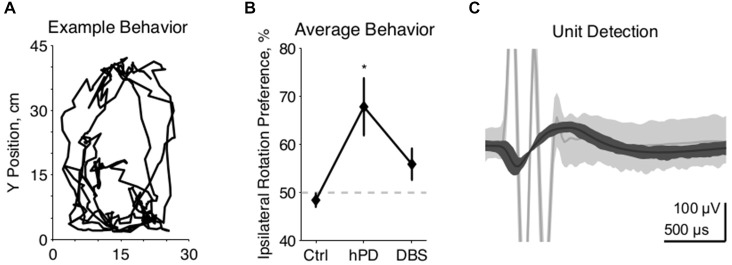
**Experimental methods. (A)** Example movements from 2 min of unrestrained rat behavior, collected 2 weeks after 6-OHDA injection to the right MFB. Rat completed multiple clockwise circles, ipsilateral to injection. **(B)** Behavioral effects of 6-OHDA injections and STN-DBS, quantified as rotation to the right divided by the integrated rotation in both directions, expressed as a percentage, mean ± SE. Asterisk denotes statistically significant difference from 50% (*p*_t-test_ < 0.05). Lesioned rats rotate preferentially ipsilateral to injection, and are thus classified as hemiparkinsonian (hPD). **(C)** Representative example of a neuronal spike unit (*dark gray*) sorted separately from a DBS artifact (*light gray*), with center lines showing the average waveforms of both clusters.

Voltage-threshold crossing segments were extracted from 500 μs before threshold crossing to 1500 μs after threshold crossing. Unit waveforms were isolated in Plexon Offline Sorter using standard techniques (Figure 1C). Large cross-channel events and stimulation artifacts, which were consistently much larger than neuronal unit activity, were invalidated. Principle components of the remaining waveforms were calculated and used to sort the waveforms into clusters via expectation maximization. When that algorithm failed to identify an obvious unit cluster, the waveforms were resorted via the valley seek algorithm. Unit clusters with an average waveform that differed qualitatively from a stereotypical extracellular action potential were manually removed. Units separated from an unsorted noise cluster and all other unit clusters at an alpha-level of 0.05 were included in subsequent analyses.

Because stimulation artifacts prevented waveform detection over ~1 ms windows every 10 ms, we analyzed data both with and without blanking 1.0 ms of activity, repeated every 10 ms. Comparing data collected under control or hPD conditions to data collected under STN-DBS conditions, qualitative differences in information metrics between analyses that did or did not include the blanking procedure were uncommon and are described in results. In general, we present information results from data analyzed without the blanking procedure. However, to correct for the simple spike frequency bias from detecting threshold crossings for only 9 ms out of every 10 ms, firing rates from STN-DBS trials were multiplied by ten-ninths.

Firing rate was found for each unit with at least 500 spike waveforms. Firing pattern entropy was estimated for the same units, from relative duration distributions of inter-spike intervals (ISIs) segmented logarithmically into five bins per decade (Dorval, 2008). From each distribution of ISIs *y*, firing pattern entropy was calculated according to the following: *H*(*y*) = – Σ_*i*_ *p*(*y_i_*) log_2_ *p*(*y_i_*), where *p*(*y_i_*) is the probability of an ISI being in bin *i*, and the entropy *H*(*y*) has units of bits/spike (Rieke et al., 1993; Strong et al., 1998).

Cross-correlations were calculated between all simultaneously recorded neurons. Unit impulse spike trains were convolved by a gaussian kernel with a 25 ms standard deviation and unit area. The waveforms were mean subtracted, and correlated with those from other units recorded simultaneously. Those cross correlation functions were compared for maximum values and periodicity.

#### Neuronal Information

Directed information was estimated for each pair of neurons recorded simultaneously from distributions of the cross- spike intervals (CSIs) binned identically to the ISIs (Dorval, 2011; Dorval and Grill, 2014). From each distribution of CSIs *x*, information directed from cell *x* to *y* was found as follows: *I*(*y:x*) = *H*(*y*) – *H*(*y|x*) from conditional entropy *H*(*y|x*) = *H*(*y*, *x*) – *H*(*y*), and joint entropy *H*(*y*, *x*) = – Σ_ij_ *p* (*y_i_*, *x_j_*) log_2_ *p*(*y_i_*, *x_j_*), where *p*(*y_i_*, *x_j_*) is the probability that an ISI is in bin *i* and the simultaneous CSI is in bin *j*. Entropy and information estimates from two dimensional, consecutive ISI and CSI distributions yielded qualitatively similar results for high firing rate pairs, but variable results for low rate pairs due to under-sampling problems associated with too few ISIs and CSIs. Thus, we present only one dimensional entropy and information estimates.

We label the information defined above as the conditional information, *I*_cond_ = *H*_naive_−H_cond_. Conditional information *I*_cond_ is necessarily non-negative, as the conditional entropy *H*_cond_ must be less than or equal to the naive entropy *H_naive_*. Problematically, for two completely unrelated spike trains given finite data sets, conditional entropy *H*_cond_ will be less than naive entropy *H*_naive_, ensuring that conditional information *I*_cond_ will be positive, despite signal independence. To correct for this positive bias, we estimated directed information as: *I*_dir_ = *I*_cond_−I_shuf_, from the shuffled information *I*_shuf_ = *H*_naive_−< *H*_shuf_ >. The mean shuffled entropy < *H*_shuf_ > was found as the average of the conditional entropy calculated from each of 100 simulations in which the ISIs *y* were kept in the same order, but the CSIs *x* were reordered randomly (Gaudry and Reinagel, 2008).

The shuffled information quantifies the mean information that would be estimated from the ISI and CSI distributions given true independence of cells *x* and *y*. Subtracting this shuffled information from the conditional information yields a bias-corrected, directed information *I*_dir_. For a set of truly independent neurons, the distribution of bias-corrected information would be gaussian with zero mean. When this process was applied to our data, the resulting distribution was nearly gaussian, with a prominent mode near 0 bits/spike but a long tail in the positive direction. This long positive tail comprises pairs with substantial directed information, while the negative side represents the left-half of the zero-mean distribution of truly independent pairs. Thus, the left-half of a gaussian curve was fit to the negative side of the bias-corrected distribution, yielding the expected standard deviation if all pairs were truly independent. Directed information between pairs at least 1.645 standard deviations above 0, and thus highly unlikely to be independent, were considered significantly informative (α = 0.05).

#### Local Field Potentials

In four animals, field activity was recorded simultaneously with unit activity and low pass filtered below 675 Hz. Power spectral densities were calculated for all LFPs via standard methods. To co-analyze LFPs with single unit activity, spike times were converted to unit impulse trains. Spectral cross bicoherence was computed for simultaneously recorded LFPs and spike trains on the same channels, according to standard methods. This cross bicoherence reports the strength of phase coupling between all frequencies in the LFPs with all frequencies in the spike trains, normalized from 0 to 1. Qualitative effects regarding beta-beta bicoherence were similar in all animals, but one animal was excluded from the population average figure (Figure 4, right) due to a substantially lower bicoherence across all frequencies.

#### Statistical Comparisons

**Figure 2:**
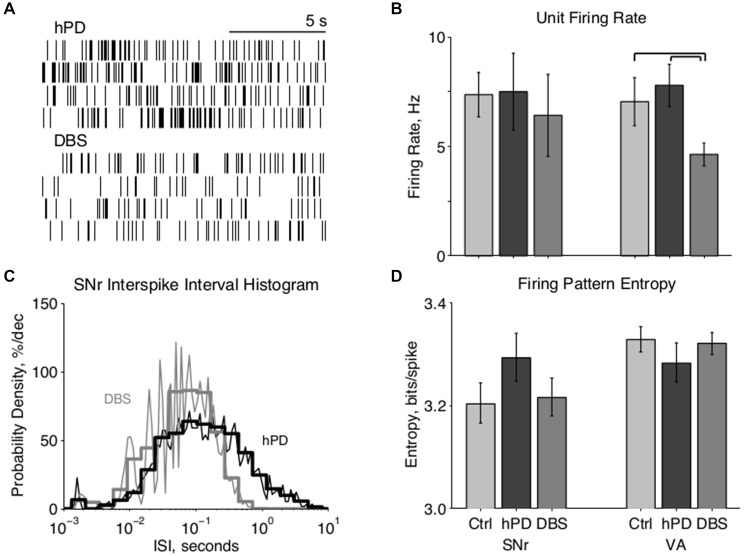
**Single unit firing pattern activity. (A)** Representative rastergrams of four consecutive 15 s intervals recorded from the same VA neuron in hPD and STN-DBS conditions, firing at ~8 Hz and ~3 Hz, respectively. **(B)** Population firing rates of single units in SNr and VA in all conditions, mean ± 50% c.i. Unit rates ranged from ~1 Hz to over 50 Hz, and this extreme variability nullified statistical differences between most conditions. In VA, however, STN-DBS did reduce firing rates significantly from healthy and hPD levels (*p*_bca_ < 0.001). **(C)** Representative fine (*thin lines*, 25 bins/decade) and coarse (*thick lines*, five bins/decade) ISI histograms of an SNr neuron in the hPD and STN-DBS conditions. The wide distribution in the hPD case is regularized via entrainment to 100 Hz DBS, as shown by ISI peaks at 10 ms and subharmonics thereof, and a narrowing of the full distribution. **(D)** Population firing pattern entropy estimated from coarse (five bins/decade) ISI histograms, mean ± 50% c.i. A trend toward SNr entropy increase in the hPD condition did not reach significance with respect to either healthy (*p*_bca_ = 0.06) or STN-DBS (*p*_bca_ = 0.07) conditions.

**Figure 3:**
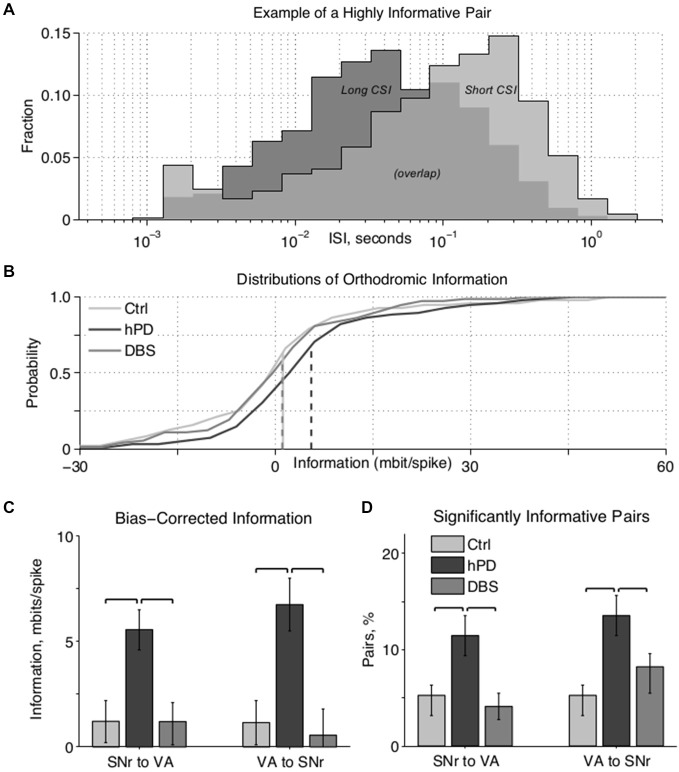
**Paired unit directed information. (A)** Example ISI histograms of one VA neuron, when a particular SNr neuron has (*light gray*) or has not (*dark gray*) spiked in the past second. A spike in the SNr cell alters the ISI histogram of the VA cell, meaning that the VA spike train carries information about the SNr spike train. **(B)** Cumulative information distributions of all SNr to VA pairs directed orthodromically. Control and STN-DBS distributions have negligible medians (i.e., at zero information probability ≈0.5) and means (*dashed lines*), which happen to overlap at ~1 mbit/spike, indicating that randomly selected cell pairs do not transmit significant information, in the hPD condition, however, the distribution has a substantially greater median and mean, indicating that many pairs do transmit information. **(C)** Bias-corrected information between all pairs in both directions, mean ± 50% c.i. Directed information was increased by hPD induction, and decreased by therapeutic STN-DBS; significance noted with black overscores (*p*_bca_ < 0.05). **(D)** Percentage of pairs in each condition transmitting significant information quantified as at least 1.645 standard deviations (i.e., *p* < 0.05) above 0, percentage ± 50% c.i. More pairs transmitted significant information in the hPD compared to the other conditions; significance noted with black overscores (*p*_bca_ < 0.05).

Where statistical significances are associated with results—i.e., Figures 1B, 2B,D, 3C,D the relevant statistical test is abbreviated in the text. Summary values of approximately gaussian-distributed results are presented as mean plus or minus standard error, mean ± SE. Summary values of non-gaussian results were found from 100,000 bootstrapped populations resampled with replacement, presented as means spanning the 25th to 75th percent confidence intervals, mean ± c.i. Statistical test abbreviations: anova = analysis of variance; *t*-test = student *t*-test; bca = bootstrapped confidence assessment.

## Results

The following includes results from five rats that developed hPD symptoms alleviated by STN-DBS.

### Model Validation

Motor symptoms of hPD were assessed from video analysis of unrestrained movements in 1 h behavioral trials (Figure 1A). Rats turned more ipsilaterally than contralaterally in the hPD condition (*p*_t-test_ < 0.05), and exhibited no directional preference in either control or STN-DBS conditions (Figure 1B). Further, there was a behavioral effect of condition (*p*_anova_ < 0.05), with rats rotating more ipsilaterally than contralaterally in the hPD condition (*p*_t-test_ < 0.05). These data summarize the responses of four of the five rats; a fifth rat had symptoms quantified via free rotation counting under dextroamphetamine injection and had similar results to those evaluated with the video-tracking system.

### Single-Unit Activity

We recorded extracellularly from SNr and VA during unrestrained behavior in all conditions—healthy, hPD, and with STN-DBS—and isolated individual unit action potentials offline (Table 1). Firing rates of individual neurons varied greatly and standard deviations were large in both anatomical locations under all conditions (Figures 2A,B). No significant changes were noted between conditions in the SNr. However, the mean firing rate in VA did depend on condition (*p*_anova_ < 0.05), significantly reduced in the STN-DBS condition (*p*_bca_ < 0.001), relative to both healthy and hPD conditions. Distributions of ISIs for each unit were compared between the hPD and DBS conditions (Figure 2C). With a fine temporal resolution (25 bins per decade), neurons in the DBS condition exhibited strongly multimodal distributions in both SNr and VA (Figure 2C, *thin lines*), with peaks at 10 ms and the subharmonics thereof (i.e., 20, 30 ms, etc). This regularity follows from entrainment of the neuronal activity to the 100 Hz DBS. However, for units with lower firing rates, 15 min trials did not yield enough data to use this high of a temporal resolution. To include all neurons under identical analytical conditions, we constructed all distributions with a lower resolution of 5 bins per decade (Figure 2C, *thick lines*). In the example, note that the 10 ms intervals are no longer visible, but that the STN-DBS distribution is substantially narrower than the hPD distribution.

**Table 1:** **Number of neuronal units and pairs**.

	Ctrl	hPD	DBS
Single units
SNr	53	26	30
VA	50	27	25
Unit Pairs
SNr → VA (Orthodromic)	95	95	73
VA → SNr (Antidromic)	95	95	73

From those distributions, firing pattern entropy was estimated for all neurons in both SNr and VA in all conditions. In VA, there were no significant changes in firing pattern entropy between conditions. In SNr, there was a trend toward increased entropy in the hPD condition relative to both the healthy (*p*_bca_ = 0.06) and DBS conditions (*p*_bca_ = 0.07); while not significant, the amplitude of these changes closely matched previously published results (Dorval and Grill, 2014). These same distributions and entropy estimates were then used to calculate directed information, as described below.

### Paired-Unit Activity

Cross correlations of neuron pairs across SNr and VA yielded no statistically significant changes in correlation between conditions. To test whether pairs of neurons were truly independent, and not merely uncorrelated, we calculated the information directed from each neuron to all other simultaneously recorded neurons.

Distributions of ISIs and CSIs were calculated from the spike times of simultaneously recorded neurons, and the directed information (bits/spike) was estimated for all simultaneously recorded pairs. The information that one cell directs toward another can be quantified as the amount by which the firing activity of the first cell modifies the activity of the second cell (Figure 3A). In this example, when an SNr cell (not shown) has not fired in the past second, the ISI distribution of a VA cell has a mode of ~40 ms (dark gray), indicating rapid firing. In contrast, when that SNr cell has fired in the past second, the ISI distribution of the VA cell has a mode of ~250 ms (light gray), indicating slow firing. Thus, by observing only the output of the VA cell, one can predict the recent activity of the SNr cell; in other words, the VA cell conveys information about the SNr cell.

After correcting for undersampling bias, the negligible information pairs are expected to constitute a zero-mean, approximately gaussian distribution. Thus, highly positive information pairs that do not fit under the gaussian curve (Figure 3B, *right-hand tail*) do not come from the zero-mean distribution, and thus convey directed information. Directed information between regions varied with condition (*p*_anova_ < 0.05) in both directions (Figure 3C). Directed information increased between the healthy and hPD conditions, orthodromically from SNr to VA (*p*_bca_ = 0.005) and antidromically from VA to SNr (*p*_bca_ = 0.004). Further, application of STN-DBS reduced directed information in both the orthodromic (*p*_bca_ = 0.003) and antidromic (*p*_bca_ = 0.003) directions. There were no significant differences in directed information between healthy and DBS conditions.

To isolate only the pairs conveying statistically significant levels of information at the α = 0.05 level, we computed the standard deviation of the zero-mean gaussian distributions, and counted the fraction of pairs conveying at least 1.645 standard deviations worth of information (Figure 3D). In the hPD condition relative to healthy, more pairs were significantly informative in both orthodromic (*p*_bca_ = 0.017) and antidromic (*p*_bca_ = 0.008) directions. In the DBS condition relative to hPD, fewer pairs were significantly informative in the orthodromic (*p*_bca_ = 0.006) and antidromic direction (*p*_bca_ = 0.05). There were no significant differences between healthy and STN-DBS conditions, in either direction.

The above comparisons were repeated with an imposed 1.0 ms blanking period to simulate the artifact in healthy and hPD trials, and qualitative results were mostly unchanged with or without blanking. The one exception was that the reduction in the number of informative antidromic pairs from hPD to STN-DBS lost significance with blanking (*p*_bca_ = 0.16). However, the result that STN-DBS reduces antidromic information is still supported with blanking, through the reduction in the mean VA-to-SNr information from hPD to STN-DBS (*p*_bca_ = 0.03).

### Unit-Field Coherence

**Figure 4:**
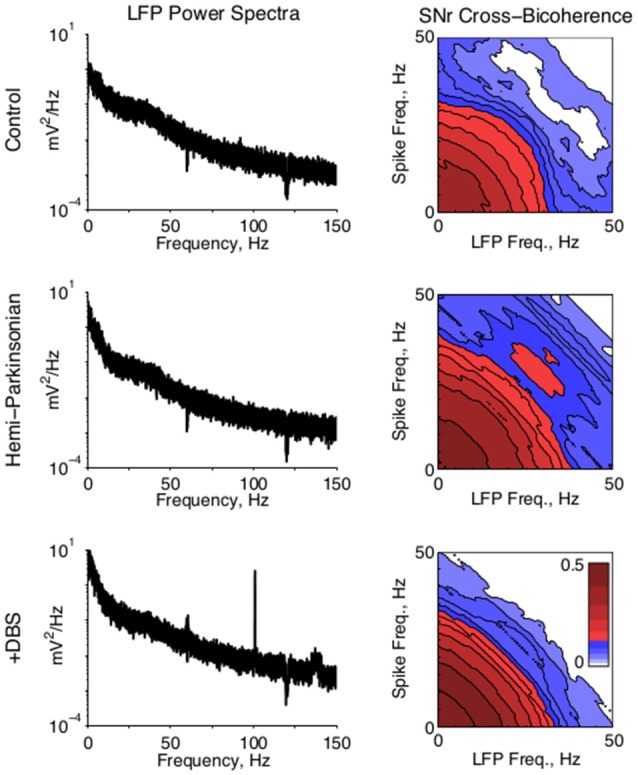
**Local field potential activity. (Left)** Representative LFP power spectra from SNr in all conditions. A strong 100 Hz peak in the STN-DBS condition reflects the stimulation frequency. **(Right)** Population average of the cross bicoherence between the unit activity and LFPs recorded on the same electrode in SNr, for all conditions. A prominent peak indicates strong beta-beta bicoherence centered at ~28 Hz in both unit and LFP frequency. Cross bicoherence at higher frequencies was generally elevated in hPD condition relative to control, suggesting stronger spike entrainment to higher frequency synaptic activity; and generally suppressed in the STN-DBS condition relative to hPD and control, suggesting relative independence of spiking from higher frequency synaptic activity.

The power-spectral densities of LFPs revealed very little of interest in either SNr or VA. In particular, changes in 20–30 Hz beta activity were not significant between conditions. However, the expected field activity at 100 Hz and its harmonics were observed in the STN-DBS condition in both locations, consistent with stimulation of the STN (Figure 4, *left*). Similarly, the cross correlations of LFPs recorded from different electrodes in SNr and VA did not change significantly between conditions.

To test whether local field activity was independent of unit activity, we found the cross bicoherence of each unit with the local field recorded from the same electrode (Figure 4, *right*). Unit-field cross bicoherence revealed a prominent interaction between high beta frequencies in SNr in the hPD condition in all four rats with recorded LFPs, but not the healthy or STN-DBS conditions in any of the rats. While beta power was not strong enough or enduring enough to manifest in the power spectra, the strong coherence between neuronal and LFP beta activity underscores the relevance of the 25–35 Hz frequency range to the hPD condition.

## Discussion

The hallmark motor symptoms of PD are driven by changes in basal ganglia neural activity. Early models of PD focused on neural firing rates, predicting that symptoms resulted from over-inhibition of the thalamus by the outputs from the basal ganglia (Bergman et al., 1994; Mink, 1996; Molnar et al., 2005). However, firing rate models of basal ganglia neural activity are not adequate to explain parkinsonian motor symptom severity, as DBS can actually increase the firing rates of neurons that inhibit the thalamus (Anderson et al., 2003; Bar-Gad et al., 2003; Hashimoto et al., 2003). Several therapies—specifically medication with levodopa, stereotactic ablation of the STN, and high frequency DBS of the STN or internal globus pallidus—alleviate motor symptoms, but these therapies each modify neuronal activity in distinct ways.

Given the variety of neuronal responses to disparate therapeutic interventions, it is unsurprising that the motor symptoms of PD manifest from changes in firing patterns, rather than merely changes in firing rate (Wichmann and DeLong, 2003). In this work, we began with the hypothesis that these changes in firing pattern constitute changes in neural information directed between neurons and nuclei. We demonstrated that lesioning the rat SNc increased information transmission between the SNr and VA, in both the orthodromic (i.e., anterograde) and antidromic (i.e., retrograde) directions, and that STN-DBS decreased information transmission back to levels similar to those seen prior to 6-OHDA administration.

Our results support the hypothesis that parkinsonian motor symptoms result from the passage of extraneous information from the ventral basal ganglia to thalamic relay neurons (Grill et al., 2004). Neuronal information transmission is bounded by neuronal firing pattern entropy, and computational studies have suggested that increased entropy in the globus pallidus, upstream from the SNr, induces errors in information transmission through the thalamus (Rubin and Terman, 2004; Guo et al., 2008; Dorval et al., 2010). While some previous work has focused on the covariance of entropy with PD symptoms (Alam et al., 2012; Lindemann et al., 2013), the reported entropy changes between conditions have been small, especially in the SNr or GPi (Dorval et al., 2008; Dorval and Grill, 2014). In the present study, entropy changes were not significant, although the general trends in SNr entropy were consistent with the reports cited above.

Despite small and insignificant changes in entropy, however, changes in information transmission were large and robust. Directed information between SNr and VA was elevated in hPD, relative to both the healthy and hPD with STN-DBS conditions. Conceptually, increases in orthodromic information transmission might be presumed as behaviorally beneficial, but recent work in non-human primates supports that basal ganglia information transmission is actually reduced by effective DBS (Agnesi et al., 2013). Furthermore, the motor symptoms of parkinsonism are not beneficial, so information increases associated with them are likely detrimental to well being. Thus, this additional information should be classified as pathological.

The pathological information in the parkinsonian condition may dictate symptoms by deleteriously modulating thalamic activity. However, the nature of this pathological modulation remains poorly understood. Signals from basal ganglia could constitute either of the following: active information that commands symptoms, or passive information that generates symptoms merely by interfering with downstream neural processing in thalamus or motor cortex. Computational studies report that prototypically parkinsonian activity from basal ganglia drives thalamic neurons to make relay errors (Rubin and Terman, 2004; Guo et al., 2008), and that the error rate increases with basal ganglia firing pattern entropy (Dorval et al., 2009). Recent studies in human DBS patients support the hypothesis that these thalamic errors passively interfere with healthy activity, rather than actively dictating symptoms. In particular, presenting increasingly entropic DBS to basal ganglia or thalamus exacerbated symptoms of PD (Dorval et al., 2010) or essential tremor (Birdno et al., 2008), respectively. The DBS signals in those studies were pseudo-random pulse trains, and thus could not have been generating meaningful commands to actively drive symptoms.

The presented analyses are subject to limitations. Our estimates of information use only the most recent ISI and CSI in their calculations. While this approach accounts for simple statistical variations like changes in firing rate, it may ignore complex dependencies that integrating over the last several CSIs could reveal. However, each additional ISI or CSI adds another dimension to the information space, dramatically increasing the data needed to estimate it (Dorval, 2008). Further, while supporting the idea that information metrics can be used to distinguish symptomatic from asymptomatic conditions, neuronal information would be difficult to measure from clinical populations. To improve DBS therapy, researchers are seeking biomarkers of PD symptom severity that can be measured in real time (Thompson et al., 2014). Thus, we extended this work to examine LFP activity relevant to information processing.

While beta activity is prominent in dorsal STN and motor cortex of parkinsonian animals, the strength of beta power is highly dependent upon behavioral state (Lehmkuhle et al., 2009). Recordings in human patients have found beta power progressively attenuated from dorsal STN, through ventral STN, and into SNr (Alavi et al., 2013). Recordings in the present study were collected from unrestrained rats who alternated between bouts of movement and bouts of rest. We did not find statistically significant beta power in field potentials collected from either SNr or VA. Further, single-unit action potentials were not particularly beta-rhythmic, even in the hPD condition.

However, the cross bicoherence between LFPs and simultaneously recorded unit activity in SNr revealed the appearance of a robust beta-beta peak in the hPD condition. This finding supports previous work that some beta coherence—which is the diagonal of our cross bicoherence—in SNr LFPs may be generated locally (Alavi et al., 2013). Further, beta-beta coherence was suppressed by therapeutic STN-DBS (Figure 4). This work supports the idea that beta synchronization throughout the basal ganglia gates parkinsonian symptoms (Brittain et al., 2014). Since LFPs likely report an integration of spatial synaptic activity, we observe that unit spike times were coherent to local synaptic activity at beta frequencies, even though beta power was not significantly larger than power in lower (e.g., alpha) or higher (e.g., gamma) frequency ranges. These findings support that a PD-associated predilection for beta entrainment is involved with the PD-associated increases in directed information. These ideas will be thoroughly explored in future work.

We do not expect that we recorded from any of the same neuronal pairs in both the healthy and hPD conditions. Given the robust increases in direct information however, we hypothesize that neuronal pairs not functionally connected in the healthy condition received and transmitted signals with similar information content in the hPD condition. This could occur if neurons in different regions became highly coherent to a particularly widespread input, as we report for beta activity (Figure 4). A recent paper showed increases in phase-amplitude coupling between beta and 250–350 Hz activity with increasing PD severity in a non-human primate model (Connolly et al., 2015). Together these studies suggest a PD-associated loss of channel independence, meaning that parallel projections from basal ganglia to motor thalamus carry redundant information in the PD condition. Even if the information between any two neurons increases with PD, if that information is redundant with information between other pairs, the net information could be reduced (Schneidman et al., 2003, 2011).

Supporting the hypothesis that PD is associated with a large increases in neural signal redundancy, directed information was similar in orthodromic and antidromic directions. Antidromic information could reflect connectivity from VA through putamen back to SNr, but more likely reflects global levels of redundant information. Recent computational work supports this interpretation, suggesting that irregular firing patterns in PD is a network phenomenon of the cortico-basal ganglia-thalamo-cortical loop, and that DBS suppresses these irregular firing patterns through orthodromic and antidromic activation (Santaniello et al., 2015). Future efforts recording from many neurons simultaneously could be used to test this hypothesis concretely. Regardless of redundancy, the hPD-associated increase and DBS-associated restoration of directed information support the notion that fewer independent channels exists in the parkinsonian condition.

A loss of channel independence through motor pathways of the basal ganglia might constrain the behaviors that a parkinsonian individual could express. Somatotopic mappings for example—as present throughout motor basal ganglia (Nambu, 2011)—are organized by musculoskeletal, anatomical relations. Thus, two independent neural pathways through basal ganglia associated with antagonistic muscles are anatomical neighbors. Increased information between neighboring neurons in antagonistic pathways would reduce the independence of the antagonistic muscles, as seen in the hallmark parkinsonian symptoms of tremor and rigidity. Similarly, loss of channel independence would reduce the ability of the basal ganglia to fulfill its action selection role (Frank, 2006; Helmich et al., 2009), particularly if the appropriate action was contaminated by alternative actions that the basal ganglia is trying to select against. This indecision may contribute to the hypokinetic symptoms of PD.

In a rodent model of PD, information transmission from SNr to VA increased in the parkinsonian condition and was restored to near healthy levels by functionally restorative STN-DBS. This study supports the growing body of work that identifies informational metrics as useful biomarkers of neurological disorders (Trevelyan et al., 2013). The pathological information generated in the parkinsonian basal ganglia likely interferes with downstream signal processing in thalamus and motor cortex, driving parkinsonian symptoms. We hypothesize that the increased information at the neuronal pair level reflects the loss of channel independence between neighboring motor command networks, and widespread informational redundancy within the parkinsonian brain.

## Conflict of Interest Statement

The authors declare that the research was conducted in the absence of any commercial or financial relationships that could be construed as a potential conflict of interest.
